# A curated dataset of 3,477 high-resolution Grapevine (Vitis vinifera) leaf images for automated detection of Black Rot, Esca, and Leaf Blight diseases

**DOI:** 10.1038/s41597-026-07431-9

**Published:** 2026-06-03

**Authors:** Milind Gayakwad, Tahseen Mulla, Deepak Parashar, Anisa Shikalgar, Prashant Chavan, Sachin Wakurdekar, Rohini Jadhav, Amol Kadam, Priyanka Paygude, Rahul Joshi

**Affiliations:** 1 Bharati Vidyapeeth (Deemed to be University) College of Engineering, Pune, India; 2 Annasaheb Dange College of Engineering and Technology, Ashta, Shivaji University, Kolhapur, India; 3 Manipal Institute of Technology, Manipal Academy of Higher Education, Manipal, India; 4 Symbiosis Institute of Technology, Pune, Symbiosis International (Deemed University), Pune, India

**Keywords:** Plant sciences, Agriculture

## Abstract

We introduce the Grapevine (Vitis vinifera) Leaf Image Dataset (GVLiD), a carefully curated set of 3,477 annotated images of Grapevine (Vitis vinifera) leaves to catalyze research in computer vision and plant pathology. Whereas the PlantVillage and Hermos datasets, for instance, contain mainly scanned or laboratory-acquired leaves, GVLiD features vineyard *in situ* images along with detailed metadata (GPS, lighting, weather, and device model) and expert-verified annotations. To measure the reliability of the annotation, label consistency was very high (κ = 0.86–0.92; 95% CI) as assessed by inter- and intra-rater agreement. Besides Indian viticulture, the dataset also aims to support the field of foliar disease detection in precision agriculture and ML benchmarking, which face significant challenges due to variable illumination and natural leaf backgrounds under field conditions. GVLiD is intended to enable worldwide, reproducible, real-world testing of AI systems for crop disease monitoring.

## Background & Summary

Grapes (Vitis vinifera) are among the most economically valuable fruit crops globally, with India being one of the leading countries in the production and export of the fruit^1^. Indian grape-growing areas, especially in Maharashtra and other subtropical regions, are highly vulnerable to fungal diseases such as Black Rot, Esca, and Leaf Blight^2,3^. Detection of these diseases in their initial stages and accurately is the only way to protect production, minimize chemical usage, and maintain the viability of grape growing^4,5^. Machine learning (ML) and deep learning (DL) methods have been effective for the automatic identification of diseases. However, their performance is highly dependent on the existence of high-quality, diverse, and properly annotated image datasets^6,7^. Several publicly available datasets exist, with PlantVillage being the largest and most popular^8,9^, offering more than 50,000 leaf images of various crops. Despite PlantVillage has been a great resource for plant disease recognition research, the limitations of the dataset have also been pointed out: (i) the majority of the pictures are taken against a controlled, uniform background, (ii) most pictures come from temperate areas, and (iii) accompanying data such as GPS, environmental context, and acquisition details are missing^10,11^. These points hinder the applicability of models trained on such data to practical field conditions, especially in tropical viticulture.

Other projects include Hermos, a European Grapevine (Vitis vinifera) dataset, which concentrates on downy and powdery mildew; the Sugarcane Leaf Dataset from India^12,13^, which mainly focuses on monoculture crops; and VegNet, which has images of vegetable diseases with a moderate variety of classes but scarce metadata. In contrast to these materials, GVLiD stands out by integrating a dual acquisition strategy (*in situ* vineyard conditions and *ex situ* controlled backgrounds)^14,15^. Locating GVLiD in this worldwide context reveals its value. Whereas datasets such as PlantVillage have provided indispensable baseline data for concept validation, GVLiD is a timely solution to the shortage of geographically diverse, metadata-rich, and field-validated datasets^16–18^. In addition to ensuring local applicability for Indian grape farming, it serves as a test environment to investigate domain adaptation, transfer learning^19^, and machine learning model robustness across different climate zones^20,21^. As viticulture is being established in tropical and subtropical regions globally, GVLiD offers worldwide-significant insights and resources that support precision agriculture, sustainable practices, and cross-regional disease surveillance.

## Methods

### Data collection

Data were collected from 34 vineyards in Tasgaon, Sangli district, Maharashtra, India (July-November 2023). A dual acquisition strategy was followed: For each leaf, photos of both the adaxial (upper) and abaxial (lower) sides were taken. All disease labels were prepared by a highly experienced agricultural expert with more than 18 years of viticulture experience. Data acquisition was done according to a documented protocol to ensure repeatability.

#### Capture specifications

- Device models: Samsung Galaxy S21 Ultra and Redmi Note 10 5 G
- Capture angle: 20–45° relative to leaf plane
- Distance: 25–40 cm
- Lighting: sunny, overcast, shaded, dusk, and controlled LED
- Background: natural vineyard (*in situ*) and neutral grey board (*ex situ*)

Both adaxial and abaxial leaf surfaces were photographed.

**Fig. 1:**
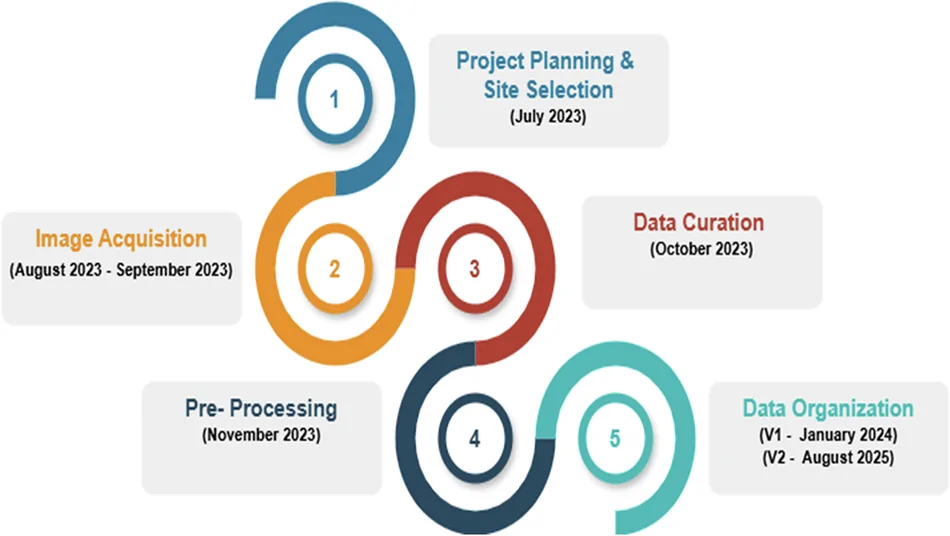
Image Acquisition and Data Organization.

The creation of specialized image datasets is an indispensable part of developing machine learning applications in agriculture, especially in areas such as precision farming and automated disease detection^22^. This dataset was created to be a top-quality resource for these purposes. The timeline showing data curation and preprocessing is in Fig. 1.

#### Data collection protocol

An agricultural expert with over 18 years of experience in viticulture verified the category of each leaf sample before imaging, ensuring label accuracy from the point of origin. A standardized acquisition protocol was followed to ensure reproducibility.

##### Data curation

Data is curated during the Project Planning & Site Selection (July 2023), Image Acquisition (August 2023 - September 2023), and Data Curation (October 2023) phases. Details are mentioned below-

Images were stored in JPEG format with a compression quality level of Q = 95, which was experimentally determined to give a good trade-off between storage efficiency and visual fidelity. For image acquisition, a dual-lighting strategy was used. *In situ* images were taken in the vineyard under different lighting conditions, including sunny, overcast, shaded, and dusk. Using this approach, the dataset obtained real-world variability such as background clutter and uneven illumination. Besides, *ex situ* images were taken in a controlled setting with a neutral gray background, and the illumination was provided by LED lights to minimize the appearance of shadows and reflections. Overall, this dual approach greatly increases generalizability: on the one hand, *in situ* data facilitates the development of models that perform well in the field; on the other hand, *ex situ* images serve as clean diagnostic references for training and benchmarking.

#### Image annotation

1. All images were labeled by an agricultural expert (Name: Mr. Avadhut Disale, Mr. Omkar Bhosale; Affiliation: Agriculture Consultant; 18 years’ experience in viticulture). Identify primary lesion type (necrotic spots, rotting margins, melanose).
2. Check lesion distribution (interveinal vs. marginal).
3. Assess presence of secondary symptoms (discoloration, pustules).
4. Cross-check with expert notes recorded at sampling.

Primary annotation was done by only one expert focusing on the domain as the main rationale; the reliability was gauged by inter- and intra-rater validations on a data subset. Although full dual annotation was not carried out, the very good levels of inter- and intra-rater agreement indicate that label consistency is reliable across the entire dataset. All the images were field-annotated by an agri-expert. Labeling was carried out via a decision tree (Figure) structure that dictated the procedure by first recognizing the main lesion type (necrotic spots, rotting margins, melanose), then analyzing lesion location (interveinal versus marginal), looking at secondary symptoms such as chlorosis, pustules, or blight patterns, and lastly, comparing with expert notes that were made at the time of sampling. This consistent annotation structure not only reduces subjectivity but also makes it easy to maintain transparency if the dataset is reused later.

### Data curation

The raw dataset initially contained 3792 images. Following a three-stage curation pipeline:

1. Integrity check: 15 corrupt files removed ( < 0.4%).
2. Quality check: 200 blurred/overexposed images excluded (5.3%).
3. Duplicate removal: 100 near duplicates identified by perceptual hashing (2.6%).

After curation, the final release contained 3,477 high-quality images, representing 91.7% of the original collection.

#### Data preprocessing (November 2023)

**Fig. 2:**
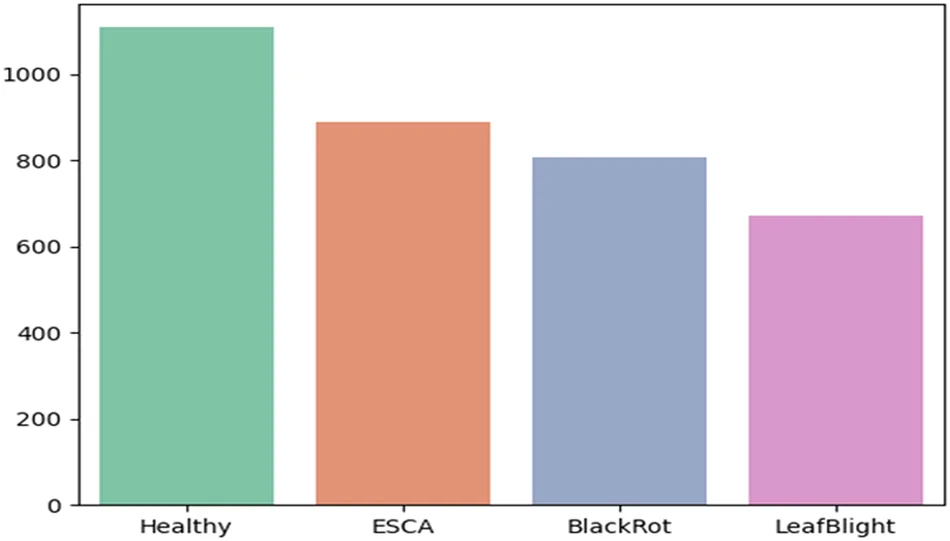
Data distribution.

Image Resizing: All images are standardized to a uniform resolution of 1080 × 1080 pixels to ensure uniformity for further processing. The data distribution is shown in the Fig. 2.

##### Data organization - final dataset

A curated, annotated, and instantly usable resource with 3,477 photos. Three different versioning stages. Originally, version 1 was the basic dataset, version 2 included the metadata, version 3 included the metadata with the details of image capture in metadata, version 4 covers the ISO standards of time of capturing the images and completeness of data, version 5 covers the image name, and sensor to capture the images of Grapevine (Vitis vinifera).

The data set shows a fair amount of class imbalance (the healthy one is overrepresented by approximately 1. 65 times). As the number of healthy images increases, considering all classes in a weighted manner helps mitigate class imbalance.

## Data Records

Data records represent a logical, programmatically reachable layout, like the data structure in Fig. 4. Data organization: The Grape Leaf Dataset contains high-resolution images of grape leaves. Every image in this dataset is a JPG file with 1080 × 1080 pixels. There are four classes in the dataset: three disease categories and one healthy-leaf category (Fig. 1). In addition to healthy leaves, the database also lists images of various Grapevine (Vitis vinifera) diseases, such as Black Rot, Esca, and Leaf Blight (Table 1). To facilitate quick access and use of the visual content, there are separate folders for each disease category. Various disease symptoms, along with natural variations across different grapevine-growing sites, were collected during field visits by experts. The directory structure used for uploading to the Mendely platform is depicted in Fig. 4. The dataset, metadata, and other files needed for data reproduction are available, and the validation script is on GitHub.

**Table 1:** Comparative Analysis.

Dataset	Crop(s)	Size (images)	Geography	Background	Metadata richness	Diseases included
**PlantVillage**^24^	Multiple crops	>50,000	Temperate, USA-based	Mostly controlled	None	38 + plant diseases
**Hermos**^25,26^	Grapevine (Vitis vinifera)	2,500	Europe	In-field	Limited	Downy, powdery mildew
**Sugarcane Leaf**^22,27^	Sugarcane	3,000	India (subtropical)	Mixed	None	Red rot, smut, rust
**VegNet**^28,29^	Vegetables	2,600	India	Mixed	Limited	15 diseases across 6 crops
**GVLiD (ours)**	Grapevine (Vitis vinifera)	3,477	India (tropical)	*In-situ* + *ex-situ*	Rich (GPS, env., device)	Black Rot, Esca, Leaf Blight

**Fig. 3:**
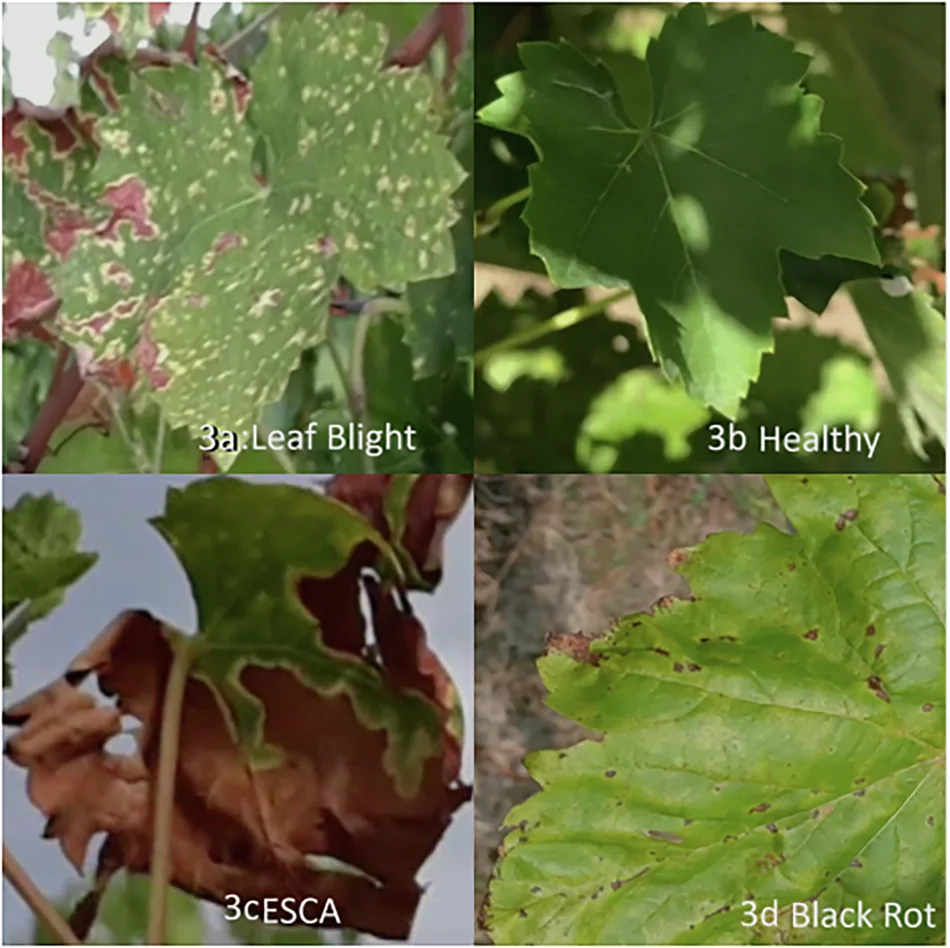
Sample Images of different Grapevine (Vitis vinifera) leaves (Unhealthy, Healthy).

Figure 3 presents some content(samples) of image(s) depicting various symptoms of the disease(s). Visualizing such features can be a useful guide to understanding how the disease works, both visually and through small-scale, first-hand reproductions of environments. Table 2 lists the location, surrounding environment, and camera type used to take the picture(s). This kind of information is a little glimpse into *ex-situ* (outside the natural location) image capturing.

**Fig. 4:**
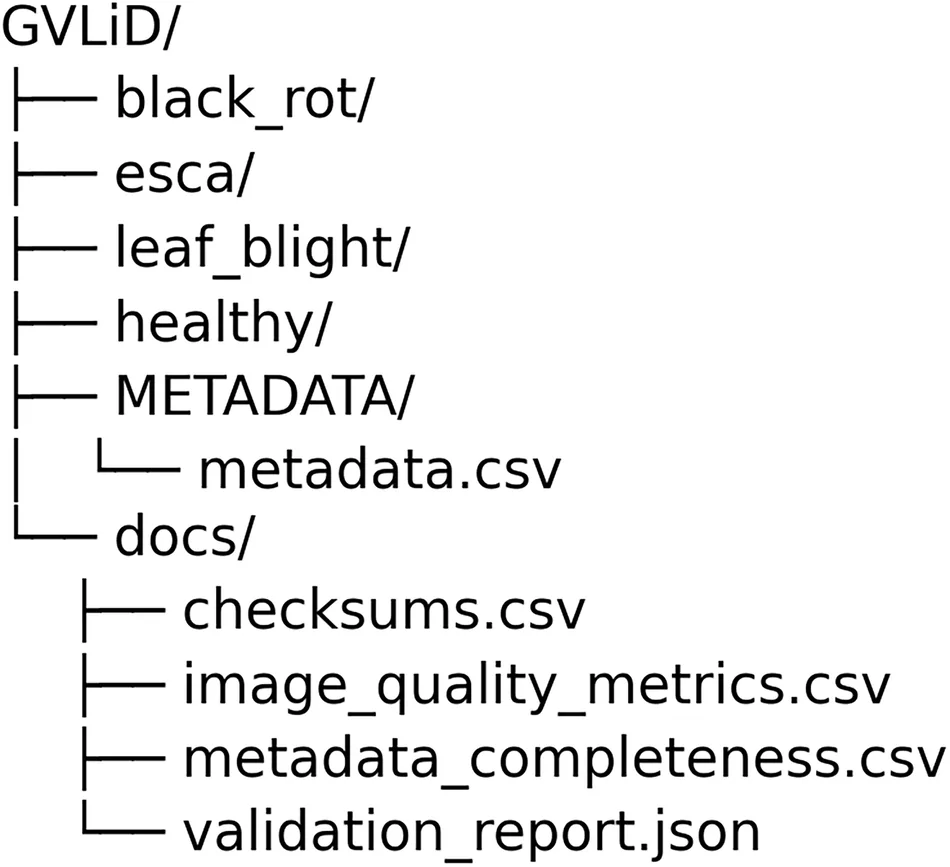
Directory Structure of the Grapevine (Vitis vinifera) Leaf Dataset.

**Table 2:** Metadata sample.

Metadata.json	Explanation
{ “Image_ID”: “IMG_0001”, “image_filename”: “black rot001.jpg”, “Location”: { “Vineyard”: “Vineyard_22”, “GPS”: { “Latitude”: 18.785238, “Longitude”: 73.994949 } }, “Environmental_Conditions”: { “Time_of_Capture”: “2023-06- 15T15:13:00 + 05:30”, “Lighting”: “Overcast”, “Weather”: “Humid” }, “Plant_Health_Status”: “Diseased”, “Disease_Type”: “Black Rot”, “Device_Specifications”: { “Device_Name”: “Redmi Note 10 5 G”, “Sensor”: “Sony IMX 582 1/2rr”, “OS”: “Android” } }	**Image_ID** (integer) — serial number indicates the image number **image_filename** (string) – Name of the image indicates the name of the disease and a unique number. **Location** **Vineyard** (string) — vineyard identifier (e.g., Vineyard_22) **GPS**. **Latitude** (float) — decimal degrees (WGS84) **Longitude** (float) -decimal degrees Time_of_Capture (ISO 8601 string) — e.g., 2023-08-15T14:35:00 + 05:30 **Lighting** (controlled vocabulary: sunny, overcast, shade, dawn/dusk, artificial) **Weather** (string) — short descriptor (e.g., humid) **Plant_Health_Status** (enum: Diseased, Healthy) Disease_Type (enum: Black Rot, Esca, Leaf Blight, Healthy) Device_Specifications Device_Name (string) — e.g., Redmi Note 10 5 G Sensor (string) — e.g., Sony IMX582 OS (string) – e.g. Android

This is the actual sample of the metadata available at the dataset organization.

The dataset is organized into three main components: **(1) Image data, (2) Metadata, and (3) Documentation/Validation files**

**Root Directory: GVLiD/**

This is the main dataset container that ensures all components required for reproducibility are available in one place.

### Image data

The GVLiD dataset structures its image data by categorizing the images of Grapevine leaves into different class-wise folders, like black_rot, esca, healthy, and leaf_blight. Each folder contains Grapevine (Vitis vinifera) leaf images at 1080 × 1080 pixels. This level of directory categorization directly corresponds to disease labels, ensuring that every image is clearly linked to a single class. Maintaining separate folders for each disease category reduces the likelihood of label errors and also helps with data loading, preprocessing, and model training. So, this dataset is very useful for tasks such as classification, benchmarking, and comparative analysis of plant disease detection.

#### Metadata

Metadata is created during the moment of taking the picture: information that does not change, like device details, is already saved, while information that changes, like exact location, time, and type of image, is input during capture. Every photo has a metadata record in JSON format. Metadata fields include vineyard ID, GPS coordinates, capture time, lighting conditions, weather conditions, device specifications, plant health status, and disease type. This approach improves reproducibility and allows for the exploration of the relationship between the environment and disease manifestation. The exact data can be found in Metadata. JSON is referred to immediately after Table 2. Metadata is written in JSON format to document parameters such as lighting conditions, plant health, and the device name.

## Technical Validation

The dataset went through a multi-step validation process.

Expert verification - The plot agronomist, physically present at the site, verified all the labels with us again, and they are 100% accurate. The image labeling was accurate, as confirmed by the feedback of the Agricultural Experts.

Balanced classes - Distributing classes evenly reduces the risk of model bias.

Image standardization: Every picture was resized to the same size beforehand. The pictures were processed to reveal the features that are needed to distinguish the disease. We have subjected GVLiD to a multi-layer inspection, including checks of dataset integrity, metadata completeness, image quality, and annotation reliability. All these inspections show that the dataset can be safely applied in machine learning, computer vision, and plant pathology research.

### Image integrity

SHA-256 checksums for each image are provided in docs/checksums.txt, allowing users to independently verify file integrity.

### Image quality assessment

To ensure consistency and suitability for automated analysis, we computed basic image quality metrics:

1. **Resolution**: All images standardized to 1080 × 1080 pixels.
2. **Compression**: JPEG, Q = 95 (empirically optimized to balance fidelity and size).
3. **Brightness**: Mean luminance = 127.3 (SD = 18.5).
4. **Sharpness**: Mean Tenengrad score = 8.7 × 10³.
5. **Blurriness**: 73 images (2.1%) flagged as potentially blurry by automated heuristics; manual review confirmed that most retained diagnostic value.

### Metadata completeness

The metadata file (metadata.json) provides rich contextual information. Completeness was quantified across 3,477 entries:

As mentioned in Table 3, the complete statistics are summarized in docs/metadata_completeness.csv.

**Table 3:** Completeness of the Metadata.

Sr_No	Field Name	Available/Total	Completeness (%)
1	Sr_No	3477	100.0
2	Plant_Health_Status	3477/3477	100.0
3	Disease_Type	3477/3477	100.0
4	Location.Vineyard	3477/3477	100.0
5	Location.GPS.Latitude	3477/3477	100.0
6	Location.GPS.Longitude	3477/3477	100.0
7	Environmental_Conditions.Time_of_Capture	3477/3477	100.0
8	Environmental_Conditions.Lighting	3477/3477	100.0
9	Environmental_Conditions.Weather	3477/3477	100.0
10	Device_Specifications.Device_Name	3477/3477	100.0
11	Device_Specifications.Sensor	3477/3477	100.0
12	Device_Specifications.OS	3477/3477	100.0

### Annotation reliability

We assessed label reliability through inter- and intra-rater agreement:

1. A random subset of 200 images was re-annotated after four weeks by the same expert (intra-rater) and by a second expert (inter-rater).
2. **Inter-rater agreement**: Cohen’s κ = 0.86, 95% CI [0.82–0.90], indicating *substantial agreement*.
3. **Intra-rater agreement**: Cohen’s κ = 0.92, 95% CI [0.89–0.95], indicating almost perfect agreement.
4. Disagreements (<5% of subset) were resolved by consensus, and approximately 3% of images in the dataset are flagged as “uncertain” in metadata.

### Class balance

To identify potential biases, we quantified the distribution of classes:

1. **Black Rot**: 809 images
2. **Esca**: 888 images
3. **Leaf Blight**: 672 images
4. **Healthy**: 1109 images

While the dataset is relatively balanced, there is a slight skew toward Healthy Images (1109), which should be considered when using the dataset. Stratified sampling is recommended, with 70% of the data for training, 15% for validation, and 15% for testing. Stratified sampling is recommended.

### Reproducibility

All validation scripts (image-quality metrics, metadata-completeness checks, and annotation-reliability calculations) are publicly available in the GVLiD GitHub repository. This ensures full reproducibility of all validation results reported here.

git clone https://github.com/MilindGayakwad/DNN

cd DNN/GVLiD

python validate_metadata.py

python compute_quality_metrics.py

This script generates a validation report including completeness statistics, missing values, and schema compliance checks.

python validate_metadata.py --input metadata.json --output report.json

#### Limitations

GVLiD dataset was acquired in Tasgaon (Sangli district) of Maharashtra and hence primarily reflects the disease symptoms and the grapevine varieties of that region and season. Therefore, if a model is trained only on GVLiD data, it is likely to perform worse on leaves from other climate zones or cultivars. GVLiD has included both *in situ* and *ex situ* photos to address domain variability to some extent; however, further geographic expansion is required to achieve worldwide generalization. Despite GVLiD offering a varied collection of real-life grapevine (Vitis vinifera) leaf images, some drawbacks still exist. For one, the dataset was gathered from a single geographical location (Tasgaon, Sangli district, Maharashtra, India), which might limit the representation of grapevine (Vitis vinifera) cultivars and viticultural environments outside this region. Two, the data were collected for one single growing season (July-November 2023), excluding seasonal variation in disease expression. Three, the pictures were taken using two different smartphone camera sensors, which could lead to slight differences in color tone and sensor properties. Though this discrepancy enhances the machine learning models’ resilience, it could also lead to device-specific biases. Finally, the dataset includes multiple disease stages but does not explicitly label the progression stage of each infection, which may be relevant for studies focusing on disease severity estimation.

## Usage Note

GVLiD is designed to facilitate ML/DL research on Grapevine (Vitis vinifera) disease detection. Its organized directory structure makes it directly compatible with frameworks such as TensorFlow and PyTorch. Researchers can explore tasks such as image classification, transfer learning, and domain adaptation. The metadata allows additional studies on environmental factors, generalization, and cross-regional disease analysis. The dataset also provides a foundation for smartphone-based viticulture applications.

## Data Availability

The dataset is available at Mendeley Data^23^.

## Code availability

The link to the GitHub project is https://github.com/MilindGayakwad/DNN.
